# Left ventricular outflow tract obstruction after transcatheter mitral valve replacement: a case report with a multifaceted approach

**DOI:** 10.3389/fcvm.2024.1431639

**Published:** 2024-08-21

**Authors:** Berenice Caneiro-Queija, Claudio E. Guerreiro, Julio Echarte-Morales, Rodrigo Estévez-Loureiro, Manuel Barreiro-Pérez, Rocío González-Ferreiro, Francisco Estévez-Cid, Juan José Legarra, Jose Antonio Baz, Andrés Íñiguez-Romo

**Affiliations:** ^1^ Department of Cardiology, Álvaro Cunqueiro University Hospital, Vigo, Spain; ^2^ Cardiovascular Research Group, Department of Cardiology, Álvaro Cunqueiro University Hospital, Fundación Biomédica Galicia Sur, Servizo Galego de Saude, University of Vigo, Vigo, Spain; ^3^ Department of Cardiovascular Surgery, Álvaro Cunqueiro University Hospital, Vigo, Spain

**Keywords:** Tendyne, left ventricular outflow tract obstruction, transcatheter mitral valve replacement, mitral annular calcification, alcohol septal ablation (ASA)

## Abstract

An 83-year-old woman was admitted to our center because of heart failure. Transthoracic echocardiography revealed severe mitral annular calcification resulting in a double mitral valve lesion. After discussion by the heart team, transcatheter mitral valve replacement with Tendyne (Abbott Structural, Santa Clara, CA, USA) was performed. Despite having a predicted neo-left ventricular outflow tract (LVOT) above the cut-off value, the patient developed clinically significant LVOT obstruction (LVOTO) refractory to medical treatment. This situation is often treated before the intervention, and dealing with LVOTO afterward can be challenging. After taking the patient's anatomy into consideration, we decided to perform alcohol septal ablation. Applying a combined strategy of medical treatment and intervention led to success. In this case report, we discuss this event and the strategies available for preventing and managing the condition.

## Introduction

According to recent data from the Euro Heart Survey, degenerative valvular heart disease is highly prevalent (1). Mitral annular calcification (MAC) is a degenerative age-dependent process leading to mitral regurgitation (MR) or mitral stenosis (MS). MAC is linked to cardiovascular risk factors, the female gender, and chronic kidney disease (2). Although surgery has been the gold standard treatment for mitral valve disease, patients with MAC have been associated with an increased risk of cardiac rupture at the atrioventricular junction, perivalvular leaks, or circumflex artery injury (3). Transcatheter mitral valve replacement (TMVR) techniques have emerged in recent years to overcome the challenges of MAC treatment. In this regard, the Tendyne valve (Abbott Structural, Santa Clara, CA, USA) has proved to be a feasible option in MAC patients (4). Nonetheless, several procedural concerns need to be taken into consideration, such as left ventricular outflow tract obstruction (LVOTO) or paravalvular leak (PVL) (5).

## Case description

An 83-year-old woman was admitted to our center because of overt heart failure (HF). Her medical background revealed arterial hypertension, dyslipidemia, and obesity as cardiovascular risk factors. The patient also had a history of atrial fibrillation (AF). In 2012, she underwent cardiac surgery due to degenerative aortic stenosis, and a 21-mm aortic bioprosthesis (St. Jude Medical, Minnesota, USA) was implanted.

During the diagnostic work-up, echocardiography showed mild aortic bioprosthetic dysfunction, severe MAC resulting in a double mitral lesion (moderate MS and severe MR), and severe tricuspid regurgitation. The left ventricular ejection fraction (LVEF) was preserved with significant concentric hypertrophy (LV mass of 172 g/m^2^ and septal thickness of 15 mm) (Supplementary Figure 1A). Coronary angiography evidenced chronic total occlusion of the mid-left anterior descending artery. Right heart catheterization showed a decreased cardiac output (2.2 L/min) with significant combined pulmonary hypertension (mean pulmonary artery pressure 43 mmHg, with pulmonary capillary wedge pressure 21 mmHg). Patient symptoms were mainly due to mitral valve disease. Due to the recording of high-risk scores (EuroSCORE II 15%, with society of thoracic surgeons (STS) score of 10.8% for mortality and 30% for morbidity), the heart team considered the patient to be at extreme risk for conventional intervention and decided to evaluate her for TMVR. Transesophageal echocardiography (TEE) revealed a degenerated aortic bioprosthesis with mild-to-moderate regurgitation and a degenerative MR with evidence of chordal rupture, severe MR, severe MAC, and an aortic-mitral angle (AMA) of 135° (Supplementary Figure 1B). A computed tomography (CT) scan was also performed, with an MAC score (6) of 7 points and a predicted systolic NeoLVOT of 330 mm^2^ (Supplementary Figure 2). A 29S LP Tendyne was thus deemed appropriate for the anatomy of the patient (Supplementary Video 1).

The procedure was performed under general anesthesia through a left mini-thoracotomy using a transapical approach with three-dimensional (3D) TEE imaging guidance. A standard 0.035-inch wire was inserted into the left atrium, and a balloon tip catheter was advanced to the left atrium to ensure a free chord trajectory. A 14-Fr sheath was inserted in the apex and the valve was pre-dilated with a 22 mm balloon to ensure calcium expansion (Supplementary Video 1). Then, a 34-Fr sheath was placed over the wire into the left atrium and the 29S LP Tendyne was delivered through the sheath and partially deployed in the left atrium, until the outer valve expanded to approximately 85% of the final size (Supplementary Video 2). The outer stent was aligned with the straight edge oriented anteriorly against the aortic-mitral continuum through device rotation under TEE guidance. The delivery sheath was retracted to deploy the remainder of the prosthesis in an intra-annular position. Finally, the length and tension of the tether were adjusted to optimize MR reduction and minimize the risk of device displacement. After the procedure, a mild posterior PVL was identified (Supplementary Video 3), without significant LVOTO (Supplementary Figure 2).

**Figure 1:**
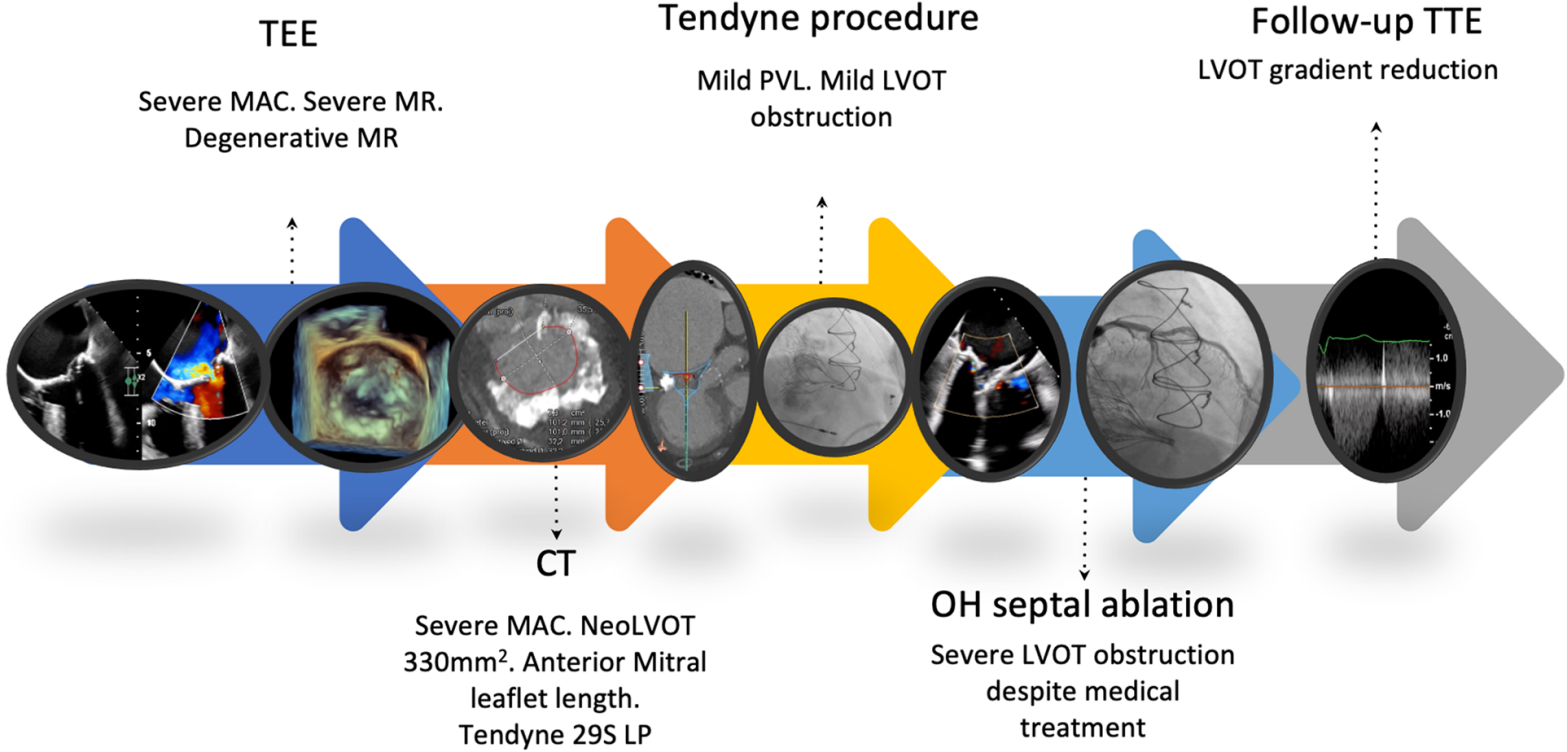
Patient timeline.

Days after the procedure, the patient exhibited poor AF rate control and HF. Transthoracic echocardiography (TTE) showed LVOTO due to contact between the Tendyne medial frame and the septal wall (Supplementary Video 4), with an initial pressure gradient of 45 mmHg. Medical treatment for the LVOTO was initially established. Beta-blockers were up-titrated according to the clinical symptoms and tolerance. The patient improved, and TTE was performed, with an LVOT gradient of 35 mmHg before discharge. However, the patient was re-admitted with HF symptoms 18 days after discharge and repeated severe LVOTO (45 mmHg was noted on TTE). After carefully reviewing the patient's history, the heart team agreed to first implant a pacemaker, and then perform alcohol septal ablation (ASA) with a 1.25 mm over-the-wire balloon in the second septal perforation artery (Supplementary Video 5). Control TTE performed 7 weeks after ASA showed a LVOT gradient of 12 mmHg (Supplementary Figure 3). At present, 6 months after the procedure, the patient presents NYHA functional class II, without signs of HF and with no further hospital admissions (Figure 1).

## Discussion

The present case report highlights the challenges associated with the transcatheter treatment of complex valvular heart disease and the broad range of interventional options we currently have to deal with them. Severe MAC is an uncommon condition, although it significantly increases the risk of mitral valve dysfunction with regurgitation and/or stenosis. Not only the increased patient age and related comorbidities, but also the associated anatomical features highlight the technical complexity of addressing this disease, which is linked to a greater risk of complications such as PVL, circumflex artery injury, disruption of the atrioventricular groove (7), conduction disturbances, and patient–prosthesis mismatch (8).

The current reported case corresponded to a high surgical risk patient of advanced age, with previous open-heart surgery and with isolated post-capillary pulmonary hypertension. To minimize the procedural risk, a stepwise approach should be taken into consideration. First, these cases require thorough discussion within a multidisciplinary heart team specialized in valve management, to properly individualize care, balancing the risk and benefit of an invasive strategy. Second, pre-procedural planning with multimodality imaging is pivotal, involving not only echocardiography but particularly also CT to assess the valvular anatomy and the extent and distribution of MAC—including the involvement of other adjacent structures—and to predict device implantation and its potential complications such as LVOTO. Initially, we estimated the LVOT-box area as described previously (9) to predict the risk of LVOTO, obtaining an area of 430 mm^2^, thus we continued the Tendyne screening process. Taking into account the CT features of mitral annulus calcium thickness, distribution, and trigone and leaflet involvement, the MAC score was seen to be 7. According to Guerrero et al., this score is associated with a 5.86-fold increase in the odds of valve embolization/migration when using a balloon-expandable valve for valve-in-mitral annular calcification (ViMAC) transcatheter aortic valve replacement (TAVR) (6). Also, mitral valve area (MVA) was close to 750 mm^2^, which fits a dedicated mitral prosthesis better rather a TAVR in mitral position. In addition, the measured NeoLVOT was 330 mm^2^, which was clearly in the theoretical safety zone, above the cut-off value of 189.4 mm^2^. This threshold demonstrated a sensitivity of 100% and a specificity of approximately 97% in predicting a post-TMVR increase in LVOT gradient of 10 mmHg or more, which according to the Mitral Valve Academic Research Consortium criteria defines iatrogenic LVOTO (10). In this context, when measuring for the Tendyne (Abbott Structural, Santa Clara, CA, USA) or Intrepid (Medtronic, Minneapolis, MN, USA) system, the minimized stent frame projection to the outflow tract would result in a slightly larger size NeoLVOT.

Surgical options for MAC-related mitral valve dysfunction are associated with operative mortality rates of 14% or higher (8). Although isolated MR could be treated with repair only, which is associated with a better prognosis and a lower rate of complications, these patients more commonly present mixed disease requiring a replacement approach (11). A range of resect and respect surgical options are available for mitral valve repair or replacement, with the former involving extensive *en bloc* resection of annular calcium, and the latter preferentially working around the calcium to avoid the complications associated with annular debridement (12). Alternative approaches like atrial-to-left ventricular valved conduits from the left atrium to the left ventricle for bypassing the mitral valve have also been described (13).

Our patient did not have an acceptable surgical risk, and the pre-procedural CT study moreover showed complete MAC, deeming her not eligible for conventional mitral valve surgery because of the increased risk for AV groove disruption. The heart team therefore opted for a transcatheter approach, choosing a Tendyne prosthesis.

In fact, percutaneous or hybrid approaches have been evaluated to try to minimize the operative risk. ViMAC approaches using a hybrid (transatrial or transapical) or a fully percutaneous strategy (transseptal ViMAC) are of interest in this scenario, but are not without their own challenges. As the anterior mitral leaflet (AML) is left *in situ*, both the transseptal and the transapical approaches are associated with a risk of LVOTO, particularly in patients with a smaller left ventricle, septal hypertrophy (14), longer or redundant anterior mitral valve leaflets, and a smaller AMA angle. In the TMVR registry, patients submitted to ViMAC TMVR had a lower overall technical success rate (62.1%) compared with valve-in-valve (94.4%) or valve-in-ring patients (80.9%), mainly because of LVOTO, which occurred in up to 39.7% of the patients. All-cause mortality was high at 30 days (34.5%) and 1 year (62.8%) (15). A meta-analysis of 13 studies involving ViMAC TMVR with balloon-expandable heart valves reported a median rate of LVOTO of 11.2%, a 3.7% incidence of transcatheter heart valve embolization, and a 4.1% incidence of moderate-to-severe PVL (16). Dedicated systems have been developed for TMVR, such as the transapical dual-stent frame Tendyne prosthesis (Abbott Structural, Santa Clara, CA, USA), which has been used in our report, with promising results according to a recent study (all-cause mortality rate of 5% and 40% at 30 days and 1 year, respectively, PVL and embolization rates of 0%, and an LVOTO rate of 5%) (17); moreover, LVOTO together with annular size are the mains reasons for screen failure with this device.

While not fully expected, in our clinical case TMVR resulted in LVOTO days after the procedure; the condition proved refractory to medical treatment and was clinically significant, resulting in decompensated HF. The position and angulation of the Tendyne toward LVOT is not always the same as it was predicted on the simulation, because the entry point can be more anterior than expected. Likewise, LV size can decrease after MR elimination and this fact can modify the relationship between the frame and the LVOT (18), and probably a combination of these two factors could influence the late development of LVOTO.

In patients with an increased risk of LVOTO, several techniques can be adopted preemptively or after its diagnosis to tackle both of the main underlying mechanisms: AML displacement and/or basal septal hypertrophy. For the former, intentional laceration of the AML to prevent LVOTO (the LAMPOON procedure) with an electrified guide wire can be an option (19). Another possibility would be balloon-assisted translocation of the mitral anterior leaflet (BATMAN). For basal septal hypertrophy, effective measures may comprise transcoronary ablation of septal hypertrophy (TASH) or the Septal Scoring Along the Midline Endocardium (SESAME) technique to perform an electrosurgical myotomy (20). In this case, ASA was successfully performed, achieving a reduction of the LVOT gradient of up to 16 mmHg. Different series report immediate improvements in gradient measurements following the procedure (21). There is a current shift in paradigm toward a more prophylactic approach to mitigate TMVR-induced LVOTO in high-risk patients (22), although this is not without pitfalls, because it increases the risk of permanent pacemaker implantation (from 16.7% to 35%) (23).

Mention should also be made of the possibility of using other TMVR devices with specific designs to prevent anterior leaflet displacement. Examples of this kind are supra-annular devices, such as the AltaValve System (4C Medical Technologies, Minneapolis, MN, USA), which could help mitigate the drawback of LVOTO, as it has a spherical nitinol frame sized to fit the left atrium, where it fixates, avoiding interaction with it. The first cases have been reported using a transapical approach, showing that it is a safe and effective device (24), and recently a small series of transseptal cases have been described, with excellent results (25).

Mitral annular calcification-related valve dysfunction is a high-risk condition associated with a poor prognosis and few simultaneously effective and safe therapeutic options. Appropriate pre-procedural risk evaluation by an interdisciplinary heart team and including multimodality imaging is mandatory to tailor therapeutic allocation to surgery, transcatheter techniques, or medical treatment only. Predicting potential hazards associated with each technique, such as LVOTO, allows the definition of preventive or bailout approaches such as TASH, LAMPOON, or SESAME. Other dedicated devices with intra-atrial fixation have been developed, limiting the occurrence of this dreaded complication. A larger body of data is needed to define the best strategy in each case for this particularly challenging patient population.

## Data Availability

The raw data supporting the conclusions of this article will be made available by the authors, without undue reservation.

## Supplementary material

Supplementary Figure 1 Section A: TTE four-chamber view showing a double mitral lesion with MAC. Section B: TEE color comparison over mitral valve in mid esophageal view. Section C: 3D mitral *en face* view with and without color.

Supplementary Figure 2 CT analysis through 3Mensio software. (**A**) Annular segmentation in systole. (**B**) Smallest estimated LVOT. (**C**) MAC analysis and AMA angle. (**D**) Anterior mitral leaflet length.

Supplementary Figure 3 LVOT gradient evolution. Section A: TTE three-chamber LVOT gradient days after Tendyne implantation. Section B: TTE three-chamber LVOT gradient after medical treatment before discharge. Section C: TTE three-chamber LVOT gradient 8 weeks after alcohol septal ablation.

Supplementary Video 1 3Mensio Tendyne 29S LP simulation.

Supplementary Video 2 Mitral valvuloplasty with Inoue balloon.

Supplementary Video 3 29S LP Tendyne deployment, TEE commissural A2/P2 X plane view.

Supplementary Video 4 LVOT and paravalvular leak evaluation after Tendyne deployment through TEE three-chamber view.

Supplementary Video 5 TTE five-chamber view with 29SP LP Tendyne medial frame touching the septal bulge.

Supplementary Video 6 Angiogram of the left anterior descending artery, showing second septal perforation artery.
