# Supplementary figures and images for: Left ventricular outflow tract obstruction after transcatheter mitral valve replacement: a case report with a multifaceted approach
Source: Front Cardiovasc Med. 2024 Aug 21;11:1431639. doi: 10.3389/fcvm.2024.1431639 (PMC11371709; doi:10.3389/fcvm.2024.1431639)

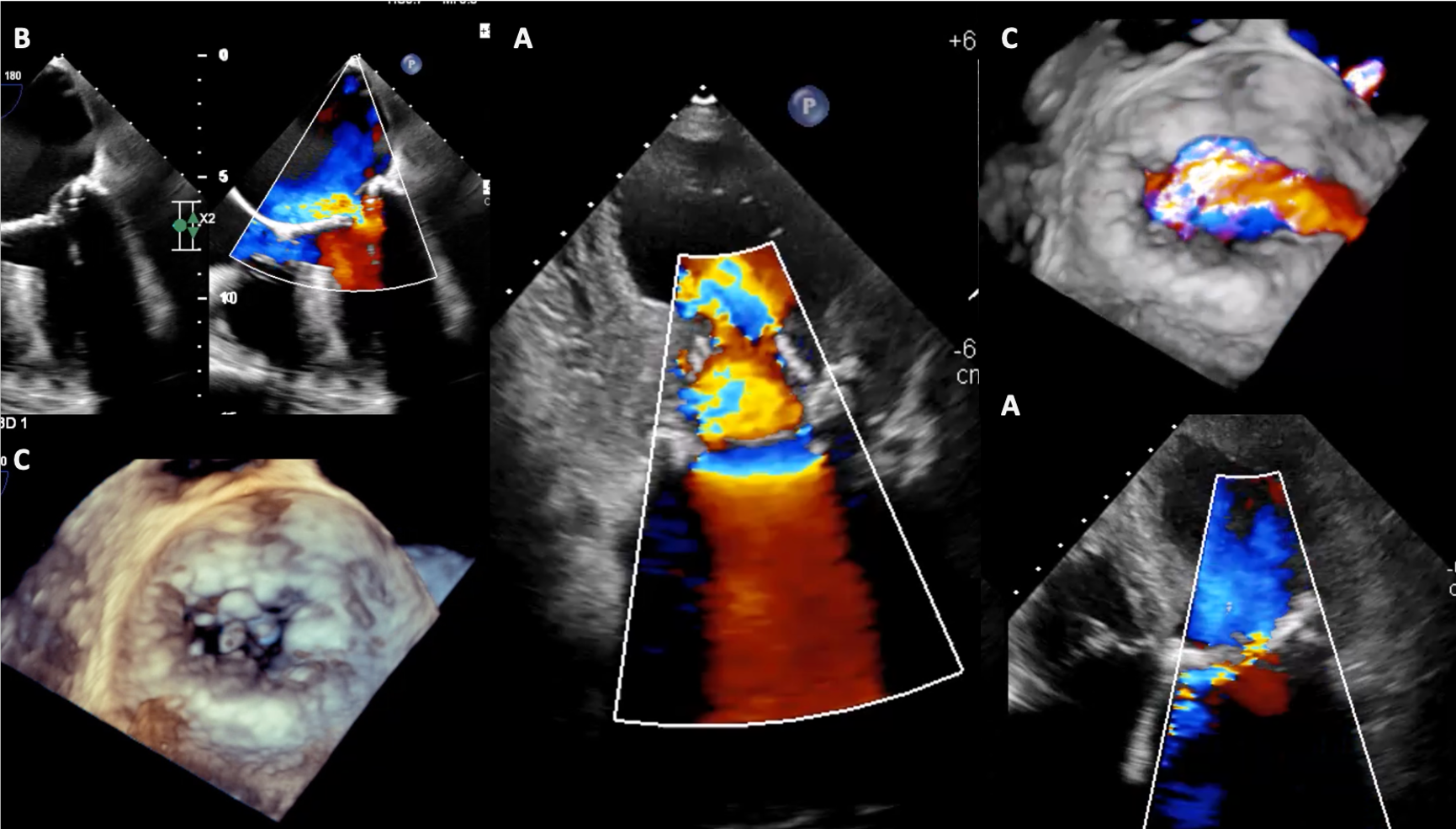

Supplement: Supplementary Figure 1 [file Image1.tiff]

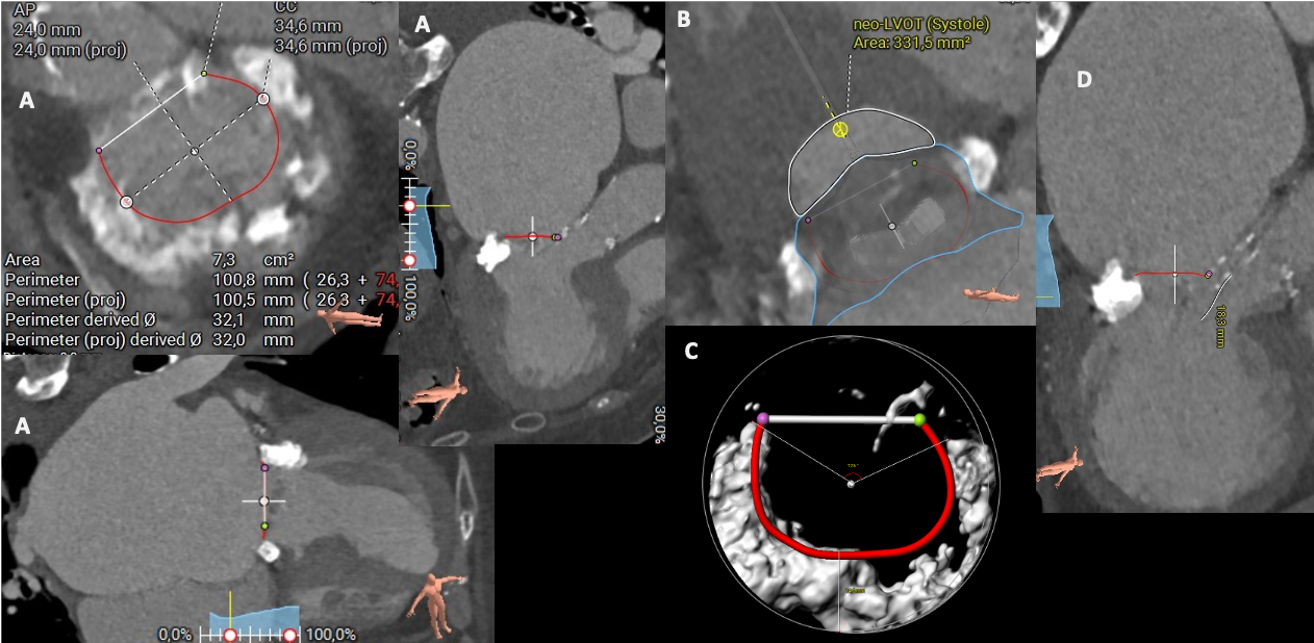

Supplement: Supplementary Figure 2 [file Image2.tiff]

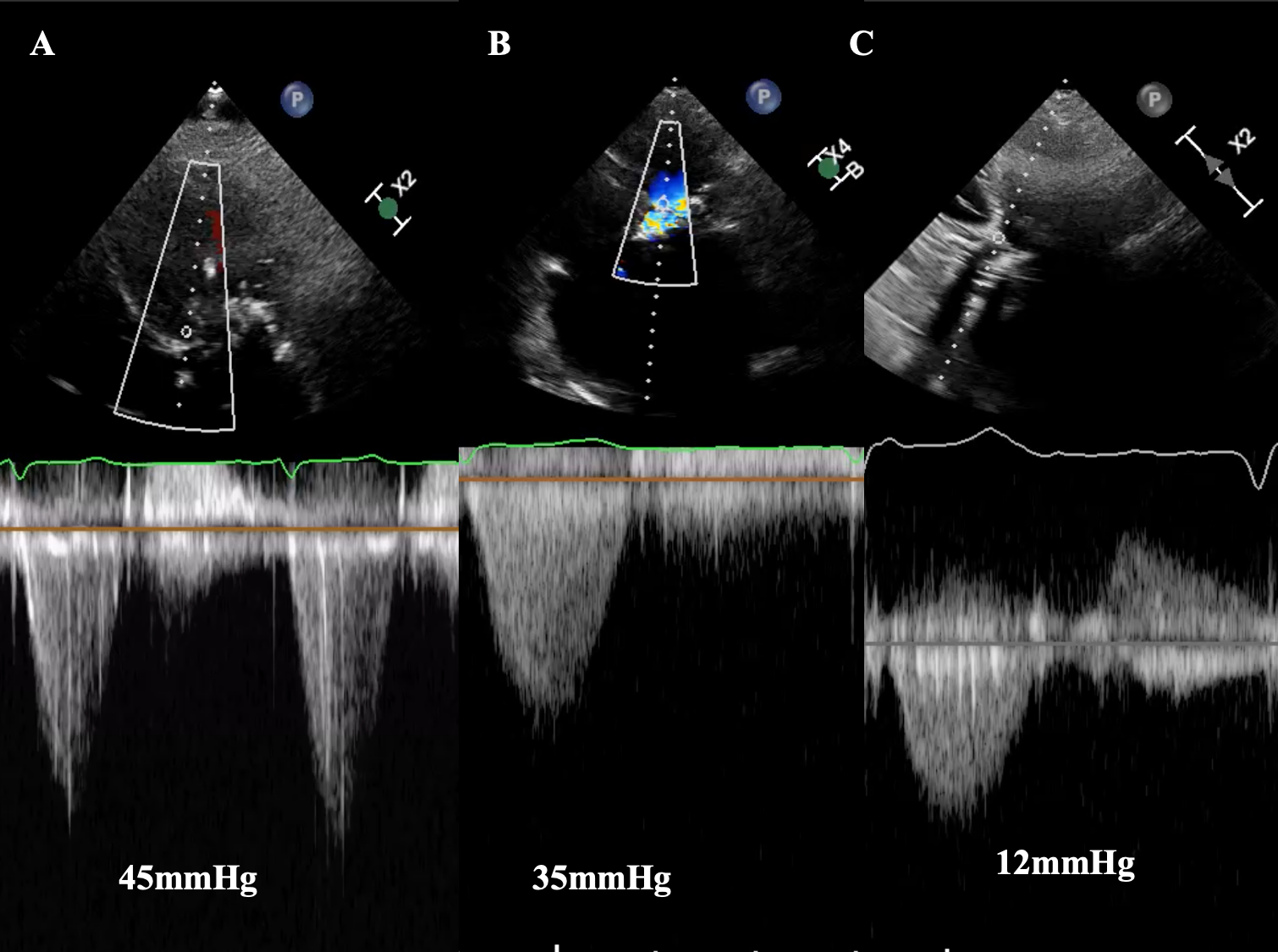

Supplement: Supplementary Figure 3 [file Image3.tiff]
